# Mammalian maxilloturbinal evolution does not reflect thermal biology

**DOI:** 10.1038/s41467-023-39994-1

**Published:** 2023-07-21

**Authors:** Quentin Martinez, Jan Okrouhlík, Radim Šumbera, Mark Wright, Ricardo Araújo, Stan Braude, Thomas B. Hildebrandt, Susanne Holtze, Irina Ruf, Pierre-Henri Fabre

**Affiliations:** 1 Institut des Sciences de l’Évolution (ISEM, UMR 5554 CNRS-IRD-UM), Université de Montpellier, Place E. Bataillon - CC 064 − 34095, Montpellier Cedex 5, Montpellier, France; 2 Staatliches Museum für Naturkunde Stuttgart, DE-70191 Stuttgart, Germany; 3 Department of Zoology, Faculty of Science, University of South Bohemia, 37005 České Budějovice, Czech Republic; 4 Department of Organismic and Evolutionary Biology & Museum of Comparative Zoology, Harvard University, Cambridge, MA 02138 USA; 5 Instituto de Plasmas e Fusão Nuclear, Instituto Superior Técnico, Universidade de Lisboa, Lisboa, Portugal; 6 Biology Department, Washington University, St. Louis, MO 63130 USA; 7 Department of Reproduction Management, Leibniz-Instiute for Zoo and Wildlife Research, 10315 Berlin, Germany; 8 Faculty of Veterinary Medicine, Freie Universität, Berlin, Germany; 9 Abteilung Messelforschung und Mammalogie, Senckenberg Forschungsinstitut und Naturmuseum Frankfurt, 60325 Frankfurt am Main, Germany; 10 Mammal Section, Department of Life Sciences, The Natural History Museum, SW7 5DB London, United Kingdom; 11 Institut Universitaire de France (IUF), Paris, France

**Keywords:** Palaeontology, Animal physiology, Phylogenetics

## Abstract

The evolution of endothermy in vertebrates is a major research topic in recent decades that has been tackled by a myriad of research disciplines including paleontology, anatomy, physiology, evolutionary and developmental biology. The ability of most mammals to maintain a relatively constant and high body temperature is considered a key adaptation, enabling them to successfully colonize new habitats and harsh environments. It has been proposed that in mammals the anterior nasal cavity, which houses the maxilloturbinal, plays a pivotal role in body temperature maintenance, via a bony system supporting an epithelium involved in heat and moisture conservation. The presence and the relative size of the maxilloturbinal has been proposed to reflect the endothermic conditions and basal metabolic rate in extinct vertebrates. We show that there is no evidence to relate the origin of endothermy and the development of some turbinal bones by using a comprehensive dataset of µCT-derived maxilloturbinals spanning most mammalian orders. Indeed, we demonstrate that neither corrected basal metabolic rate nor body temperature significantly correlate with the relative surface area of the maxilloturbinal. Instead, we identify important variations in the relative surface area, morpho-anatomy, and complexity of the maxilloturbinal across the mammalian phylogeny and species ecology.

## Abstract (web summary)

The maxilloturbinal, an area of the mammalian nasal cavity, has been proposed to play a pivotal role in body temperature maintenance. Here, the authors use computed tomographic data to show that neither corrected basal metabolic rate nor body temperature significantly correlate with the relative surface area of the maxilloturbinal.

## Introduction

The ecological and evolutionary success of mammals was highly affected by their ability to maintain a relatively environmentally-independent and stable body temperature, which allows dispersal to a wide range of habitats normally prohibited to ectotherms^1,2^. Some anatomical structures have been proposed for diagnosing and dating the origin of endothermy^1,3–9^. Among bony structures, respiratory turbinals (e.g., the mammalian maxilloturbinal and nasoturbinal) are interesting anatomical structures that may offer important insights to the origins of endothermy within extant and extinct vertebrates^3,4^. Indeed, respiratory turbinals are covered in highly vascularized epithelium that amplify surface area and offer an effective mechanism to avoid loss of internally-produced and costly heat^3,10^. During inhalation, the air is usually warmed up at contact with the vascularised epithelium of the respiratory turbinals and is simultaneously moistened by mucus glands. During subsequent exhalation, this air is cooled down by the anterior portion of the respiratory turbinals which were previously cooled down by inspired air. This process condenses water from the nasal cavity and therefore retains, on average, two-thirds of the water of the exhaled air^1,3,10–15^. Among respiratory turbinals, the maxilloturbinal is shared by all extant terrestrial mammals and it has been argued that it plays a significant role in maintaining body temperature^3,4,6,16^. Functionally, the relative size of the maxilloturbinal is related to heat and moisture conservation capacities. A convergent increase in the proportion of the maxilloturbinal has been associated with gains in thermoregulatory capacity in amphibious and aquatic mammals^17,18^.

The presence of the maxilloturbinal has been used to infer the endothermic conditions and basal metabolic rates of extinct tetrapods^1,3,4,15,19^, whereas its relative size has been argued and/or tested to correlate with body temperature and metabolic rates^6,16^. However, extant mammalian species differ in their thermal and metabolic characteristics. Diminished temperature regulation, relatively low body temperature and basal metabolic rates have been documented among marsupials, monotremes, xenarthrans, subterranean rodents, and afrotherians^20–23^, as well as some mammals that undergo different forms of heterothermy (e.g., long-term hibernation or daily torpor^24,25^). Such thermal and physiological exceptions have been hypothesized to be linked to peculiar maxilloturbinals^6^. Using a comparative three-dimensional (3D) µCT dataset of 424 skulls and unstained ethanol-preserved heads for 310 species across all major mammalian orders, we explored the anatomical diversity of the maxilloturbinal based on relative surface area, morphology and complexity. We subsequently extended the initial investigations of Owerkowicz et al.^16^ and tested the hypothesis that maxilloturbinal size reflects species thermophysiology. We specifically test the relationship between the size-corrected basal metabolic rate (cBMR) and the relative surface area of the maxilloturbinal (Maxillo RSA) as well as between body temperature (*T*_*b*_) and Maxillo RSA.

Here, we show that neither corrected basal metabolic rate (cBMR) nor body temperature (*T*_*b*_) significantly correlate with the relative surface area of the maxilloturbinal (Maxillo RSA). Instead, we identify important variations in the relative surface area, morpho-anatomy, and complexity of the maxilloturbinal across the mammalian phylogeny and species ecology. Overall, we show that the maxilloturbinal plays a moderate role in mammalian thermal biology and suggest to use other bony proxies such as the semicircular canal morphofunction to infer the endothermic conditions of extinct mammals.

## Results

### Maxilloturbinal surface area

**Fig. 1:**
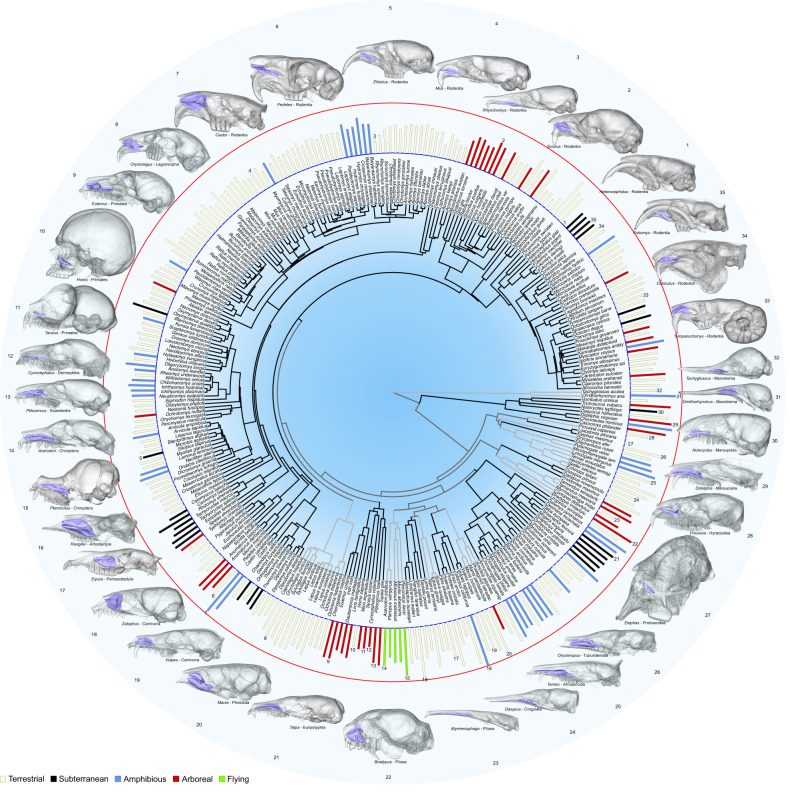
Variations of the relative surface area and shape of the maxilloturbinal between mammalian species. Barplots represent the relative surface area of the maxilloturbinal in 310 species. Blue and red circles respectively represent the minimum and the maximum values from the naked mole-rat (*Heterocephalus glaber*) and the California sea lion (*Zalophus californianus*). 3D representations of the skull and the maxilloturbinal in several species. Barplots: cream = terrestrial, red = arboreal, blue = amphibious, black = subterranean, and green = flying species. Not to scale.

**Fig. 2:**
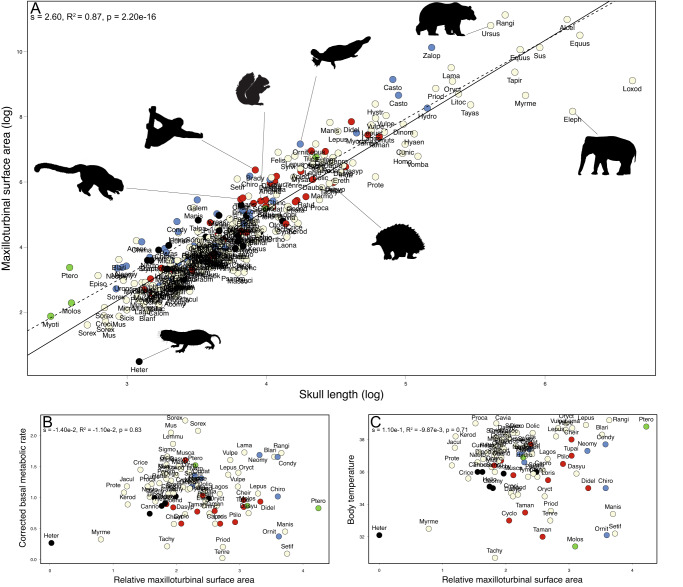
Maxilloturbinals may not always reflect thermal and metabolic conditions. **A** Log–log regression (continuous line) and PGLS (dashed line) of maxilloturbinal surface area on skull. **B** Linear regression between corrected basal metabolic rates (cBMR) and the relative surface area of the maxilloturbinal (Maxillo RSA) and (**C**) between body temperatures (*T*_*b*_) and Maxillo RSA. The *p* values correspond to the correlation based on the *stats* r package. Barplots: cream = terrestrial, red = arboreal, blue = amphibious, black = subterranean, and green = flying species. Creative commons silhouettes were downloaded from http://phylopic.org. According to the phylopic guidelines we credited T. Michael Keesey for the unmodified silhouette of *Elephas maximus* and we provided the link to the licence: https://creativecommons.org/licenses/by/3.0/.

There is a positive allometric correlation between maxilloturbinal surface area and skull length (Figs. 1, 2A, s = 2.60, R^2^ = 0.87, *p* = 2.20 10^−16^). However, some species deviate from the general trend (Figs. 1, 2A). The relative surface area of the maxilloturbinal (Maxillo RSA) is also related to species ecology and phylogenetic relationships (Figs. 1, 2A, Supplementary Table 1, Supplementary Data 1: folder 1). Maxillo RSA has a strong and significant phylogenetic signal (K = 0.04, *p* = 1.00 10^−4^; lambda = 0.98, *p* = 1.48 10^−48^). The mammalian species with the highest values of Maxillo RSA (*Castor*, *Chironectes, Galemys*, *Ornithorhynchus*, and *Zalophus*) are generally amphibious (Figs. 1, 2A, Supplementary Data 1: folder 1). They respectively have 448, 275, 329, 306, and 611% of the predicted Maxillo RSA (Supplementary Data 1: folder 1). However, the terrestrial artiodactyl *Rangifer tarandus* has the second highest predicted Maxillo RSA (463%, Figs. 1, 2A, Supplementary Data 1: folder 1). Some carnivores have among the highest values of predicted Maxillo RSA (*Felis* and *Ursus*) with 350 and 430% respectively (Figs. 1, 2A, Supplementary Data 1: folder 1), and yet *Hyaena* and *Proteles* have lower than expected Maxillo RSA, with 58 and 30% of the predicted values, respectively (Figs. 1, 2A, Supplementary Data 1: folder 1). In addition, some genera such as *Hystrix*, *Manis*, *Pteronotus*, and *Setifer* have among the highest values of predicted Maxillo RSA (286, 357, 377, and 312%, respectively, Figs. 1, 2A, Supplementary Data 1: folder 1) without any noticeable explanatory factors. *Heterocephalus glaber* was found to have the lowest value of predicted Maxillo RSA (6%, mean of 17 individuals, Figs. 1, 2A, Supplementary Data 1: folder 1). Other species with low thermoregulatory capacities such as *Bradypus* and *Tachyglossus* have 310% and 55% of the predicted Maxillo RSA, respectively (Figs. 1, 2A, Supplementary Data 1: folder 1). Elephants (*Elephas* and *Loxodonta*) have the lowest predicted Maxillo RSA after *H. glaber* (both 7%, Figs. 1, 2A, Supplementary Data 1: folder 1). However, the maxilloturbinal of adult elephants is merged with other nasal structures and is difficult to delineate (Supplementary Fig. 4). In addition, elephants have a highly modified respiratory system including the trunk which complicates comparison. However, other species with a trunk such as *Elephantulus rozeti*, present expected Maxillo RSA (78%, Figs. 1, 2A, Supplementary Data 1: folder 1). Worm-eating rodents such as *Paucidentomys vermidax* and *Rhynchomys soricoides* have some of the lowest predicted Maxillo RSA among mammals as well (16 and 22%, Figs. 1, 2A, Supplementary Data 1: folder 1). Other species with highly elongated rostrum such as *Myrmecophaga*, and *Tachyglossus* also presented low values of predicted Maxillo RSA (28% and 55%, Figs. 1, 2A, Supplementary Data 1: folder 1).

Overall, there is no significant correlation between the corrected basal metabolic rate (cBMR) and Maxillo RSA (Fig. 2B, s  = −1.40 10^−2^, R^2^ = −1.10 10^−2^, *p* =  0.83) and species with similar cBMR may have different Maxillo RSA. Conversely, species with comparable values of Maxillo RSA may have a different cBMR. Concerning the ecology, the differences observed in the maxilloturbinal surface area (*p* = 0.05), Maxillo RSA (*p* = 3.40 10^−4^), Maxillo RSA based on body mass (*p* = 0.05), skull length (*p* = 0.02), *T*_*b*_ (*p* = 0.03), and cBMR (*p* = 0.01), are significantly or marginally significantly explained by the ecology (Supplementary Table 1). However, none of the interactions between the variables as well as with the ecology are significant (Supplementary Table 1).

There is also no significant correlation between body temperatures (*T*_*b*_) and Maxillo RSA (Fig. 2C, s = 1.10 10^−1^, R^2^ = −9.87 10^−3^, *p* = 0.71) and species with similar *T*_*b*_ may have different Maxillo RSA. Conversely, species with comparable values of Maxillo RSA may have a different *T*_*b*_.

Despite the use of three different datasets, the ventilation rate did not significantly correlate with Maxillo RSA (Supplementary Fig. 5). However, since the statistical power of these linear regressions are low (based on 3 to 6 species, Supplementary Fig. 5), such results may need to be interpreted further in light of additional data that overlaps with our sampled species.

There is no consistent pattern between Maxillo RSA and the different forms of heterothermy, such as long-term hibernation, aestivation and short-term daily torpor. Indeed, *Spermophilus citellus* hibernates for 4 to 7 months^26^ and has lower predicted Maxillo RSA than *Sciurus vulgaris* (70 vs. 106%, Figs. 1, 2A, Supplementary Data 1: folder 1) which hibernates only rarely or not at all^27,28^. *Ursus arctos* is known to hibernate between 5 to 6 months per year^29^ and has lower predicted Maxillo RSA than its aquatic relative, *Zalophus* (430 vs. 611%, Figs. 1, 2A, Supplementary Data 1: folder 1). However, in this species, hibernation has little effect on the body temperature in comparison to other hibernating mammals^29^. *U. arctos* has higher predicted Maxillo RSA than the two sampled species of foxes (*Vulpes vulpes* and *V. lagopus*) that do not hibernate (430 vs. 166 and 184%^30^ Figs. 1, 2A, Supplementary Data 1: folder 1). *Cheirogaleus medius* can aestivate for the longest period of time (up to 70 days^31^) and has the same predicted Maxillo RSA as its close relative *Eulemur collaris* that is not known to aestivate (158%, Figs. 1, 2A, Supplementary Data 1: folder 1). Finally, *Glis glis* is capable of daily torpor during diet restriction and low ambient temperature, as well as hibernation and aestivation^25^. All sampled Gliridae are known to either hibernate, aestivate or are capable of daily torpor^31^. As a comparison with more phylogenetically distant species, *G. glis* has lower predicted Maxillo RSA than *S. vulgaris* (79 vs. 106%, Figs. 1, 2A, Supplementary Data 1: folder 1). Maxillo RSA is not significantly explained by the heterothermy in both cases by considering two or four categories (*p* = 0.06 and *p* = 0.27 respectively, see Methods section).

### Maxilloturbinal morphology

**Fig. 3:**
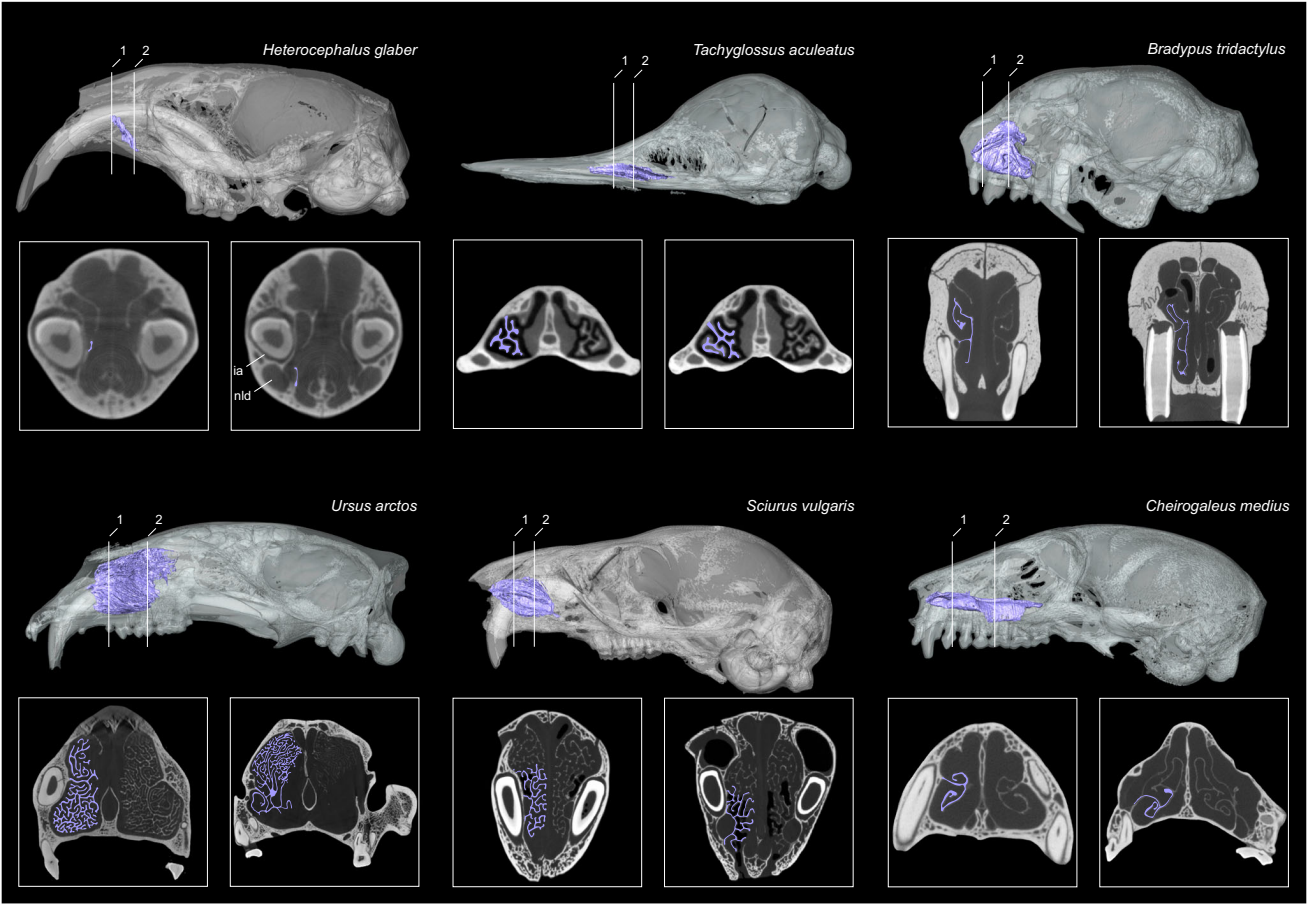
Detailed view of the maxilloturbinal in selected mammalian species with peculiar thermal and metabolic conditions or that undergo different forms of heterothermy. 3D representations and coronal cross sections of the maxilloturbinal. Not to scale. The maxilloturbinal drawings in the coronal views do not represent the actual segmentation thickness and only illustrate the maxilloturbinal.

No common morphological pattern was found among species that present particular cBMR, *T*_*b*_ or that undergo different forms of heterothermy (Figs. 1, 2, 3). As an example, *Pteronotus* and *Tympanoctomys* have a comparable cBMR but significantly differ by the anterior extension of their maxilloturbinal (Fig. 1 n 15, 33, 2B).

Among mammals, the maxilloturbinal is generally positioned ventrally in the anterior nasal cavity, however in some species where the maxilloturbinal is highly developed, it also occupies the dorsal portion. This is the case in *Castor* and *Zalophus* whose maxilloturbinal extends to the nasal roof (Fig. 1 n 7, 18). Despite its large size, the maxilloturbinal does not extend as far dorsally in *Pteronotus*, *Manis*, and *Rangifer* (Fig. 1 n 15, 20, 16). The anteroposterior position of the maxilloturbinal also varies considerably (Fig. 1, Supplementary Fig. 1). The maxilloturbinal reaches the nasal aperture anteriorly in several species, such as in *Acerodon*, *Castor*, *Didelphis*, *Pedetes*, *Procavia*, *Rangifer*, and *Tympanoctomys* (Fig. 1 n 14, 7, 29, 6, 28, 16, 33, Supplementary Fig. 1). Between the anterior part of the maxilloturbinal and the nasal aperture there is a gap in *Elephas*, *Equus*, *Fukomys*, *Heterocephalus*, *Manis*, *Notoryctes*, *Orycteropus*, *Pteronotus*, and *Tarsius* (Fig. 1 n 27, 17, 35, 1, 20, 30, 26, 15, 11, Supplementary Fig, 1). This gap is particularly developed in monotremes (*Ornithorhynchus* and *Tachyglossus*, Fig. 1 n 31, 32, Supplementary Fig. 1). It is occupied by the outer nasal cartilage and the cartilaginous margino- and atrioturbinals that are not identifiable with classical µCT^32,33^. The margino- and atrioturbinals are in most cases only slightly covered by blood vessels and mucus glands, thus, their role in heat and moisture conservation is limited^33^. The maxilloturbinal morphology typically varies with skull shape. For instance, some species with an elongated rostrum present an elongated maxilloturbinal as in *Dasypus*, *Myrmecophaga*, *Rhynchomys*, and *Tenrec* (Fig. 1 n 24, 23, 3, 25). The maxilloturbinal of some species forms a recess, which is highly developed in lineages, such as *Hystrix*, *Manis*, *Orycteropus*, and *Rangifer* (Fig. 1 n 20, 26, 16, Supplementary Fig. 2). The maximum height of the maxilloturbinal may be higher than the nasal aperture in *Castor*, *Bradypus*, *Manis*, *Ornithorhynchu*s and *Zalophus* (Fig. 1 n 7, 22, 20, 31, 18). In most species, this is the opposite pattern with a maximum difference in *Cynocephalu*s, *Elephas*, *Heterocephalus*, and *Pedetes* (Fig. 1 n 12, 27, 1, 6).

### Maxilloturbinal complexity

**Fig. 4:**
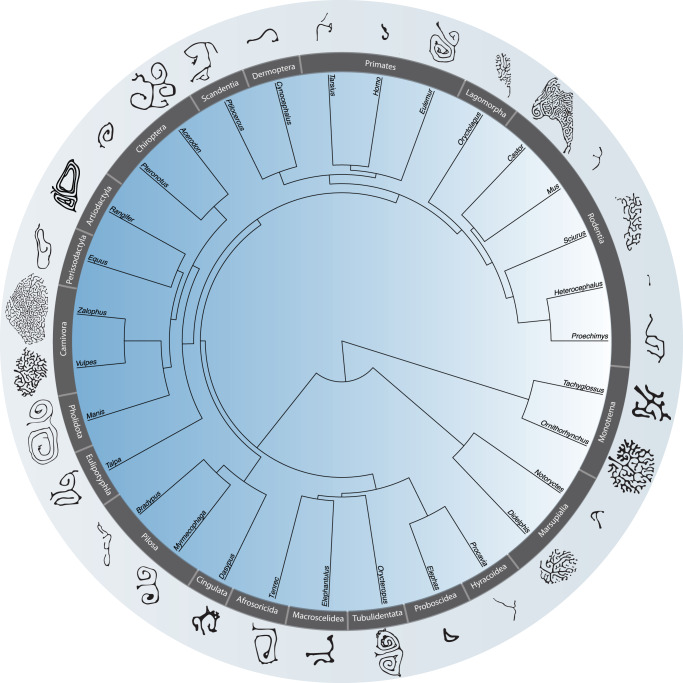
Important variations of the maxilloturbinal complexity between mammalian species. Silhouettes of the most complex area of the maxilloturbinal coronal cross section across mammalian major clades. Not to scale.

Maxilloturbinal complexity describes infolds and small lamellae that compose the turbinals (see Methods section). Maxilloturbinal complexity does not correlate with cBMR, *T*_*b*_ or different forms of heterothermy (Fig. 4, Supplementary Fig. 6). For example, *Vulpes* and *Ornithorhynchus* have a different cBMR and *T*_*b*_ but present a similar pattern of complexity with a maxilloturbinal composed of several lamellae (Fig. 2B, C, 4). Conversely, *Heterocephalu*s and *Myrmecophaga* have a comparable cBMR and *T*_*b*_, but a very different pattern of turbinal complexity (Fig. 2B, C, 4).

However, we identified that maxilloturbinal complexity varies widely among mammals and might have convergently evolved according to ecological lifestyle and/or phylogenetic relationships (Fig. 4). As an example, *Oryctolagus*, *Castor*, *Sciurus*, *Zalophus*, *Vulpes*, *Didelphis*, and *Ornithorhynchus* developed numerous lamellae and infolds resulting in a highly dendritic pattern in cross-section (Figs. 3, 4). Other species present two symmetrical and scrolled branches with a variable number of windings that originated from a single main branch (e.g., double scroll pattern, Fig. 4). This is the case in *Eulemur*, *Rangifer*, *Manis*, *Talpa*, *Myrmecophaga*, *Dasypus*, and *Orycteropus* (Fig. 4). Other species present a simple but relatively developed lamella such as *Homo*, *Cynocephalus*, and *Elephas* (Fig. 4). Lastly, when present, the maxilloturbinal of *H. glaber* is a vestigial lamina that is anteriorly attached to the medial side of the incisor alveolus (ia, Figs. 3, 4). Posteriorly, this lamina extends ventrally and merges with the canal housing the nasolacrimal duct (nld, Fig. 3).

## Discussion

### Origins of endothermy and synapsid turbinals

We have demonstrated that neither the corrected basal metabolic rate (cBMR) nor body temperature (*T*_*b*_) significantly correlates with the relative surface area of the maxilloturbinal (Maxillo RSA, Fig. 2B, C, Supplementary Fig. 3A, B). These results challenge the hypothesis positing that respiratory turbinals reflect the thermal and metabolic physiology in tetrapods and especially in mammals^1,3,4,6,16^. Indeed, an increase in metabolic rate and aerobic activity has been linked to the origin of endothermy, implying higher ventilation rate as well as water and heat loss (reviewed in^6^). Maxilloturbinals prevent water and heat loss and thus have been hypothesized to be potential osteological evidence for the origin of endothermy among tetrapods^6,16^. Given that *T*_*b*_ is a valid proxy for endothermy (e.g.^9^, and see Methods section), maxilloturbinal relative surface area could potentially be correlated to *T*_*b*_.

It was also hypothesized that respiratory turbinals may have originally been selected for heat dissipation and brain cooling then later exapted for heat and moisture conservation^16^. In addition, and depending on activity levels and ambient temperature, the respiratory turbinals of non-mammalian cynodonts may have had a dual function in heat conservation as well as in heat dissipation^6^. Some fossil evidence came from the late Permian therocephalian *Glanosuchus* (~261 Mya) bearing bony scars on the lateral wall of the nasal cavity that were interpreted as indicating the attachment area for respiratory turbinals^3,4^. Hillenius^3,4^ infers that *Glanosuchus* may be the earliest known tetrapod being endothermic and, therefore, possessing significant internal thermoregulatory capacities, however, therocephalians have been demonstrated to be ectothermic^9^. Similar bony scars were also found in the cynodont *Massetognathus*^6^, a more derived stem-mammal, that despite having relatively high thermo-motility indices when compared to other cynodonts, is ectothermic^9^. Analogously, the dicynodont *Lystrosaurus* was inferred to have potential cartilaginous maxillo- or naso-turbinals that were proposed to be evidence of endothermy in the species^19^, but anomodonts also have semicircular canal biomechanics conforming to an ectothermic status^9^. These results point to two possible outcomes: either the scars in the nasal cavity were not for anchoring the turbinals, or if there were turbinals in these taxa they did not have a thermoregulatory function originally.

### Is there a correlation between metabolism, body temperature and maxilloturbinal surface area?

Although it was previously reported that there is a correlation between field metabolic rates and corrected respiratory turbinal surface area, we show this is probably a result of methodological issues^16^. Because it is known that BMR is significantly correlated with field metabolic rates (FMR^34^), such discrepancy between our and Owerkowicz et al.^16^ results is likely explained by the small sample size of their study based on ten species only. A further potential explanation is that they used histological sections instead of 3D X-ray micro-computed tomography (µCT) data to quantify turbinal bone surface area. µCT provides a complete three-dimensional view of these complex structures, thus it is more accurate. Finally, in their study, they also included the nasoturbinal while we focus on the maxilloturbinal. Indeed, among mammalian orders the epithelial cover of the nasoturbinal is quite variable and for some, it includes a portion covered with olfactory epithelium that might be involved in olfaction^33,35–39^.

Hillenius-Ruben’s hypothesis posits that the presence of the maxilloturbinal is indicative of endothermy^3,4^. Along these lines, for example, *H. glaber* has a vestigial maxilloturbinal reflecting the thermal and metabolic conditions of the species (Figs. 1, 3, 4). This species has the lowest value of predicted Maxillo RSA (6%, mean of 17 individuals, Figs. 1, 2A, Supplementary Data 1: folder 1). Coincidently, the naked mole-rat is a poorly thermoregulating endotherm with low BMR and has been described by some authors as the only known obligatory poikilotherm mammal (e.g.^9,22,40–43^, but see^44^). However, some species among marsupials, monotremes, xenarthrans, and subterranean rodents also have low body temperature and/or low BMR and are poor temperature regulators^20–23,45^. Unlike *H. glaber*, these species retain a well-developed maxilloturbinal. *Bradypus* has among the highest values of the predicted Maxillo RSA in our sample (310%, Figs. 1, 2A, Supplementary Data 1: folder 1), whereas *Tachyglossus* has intermediate values (55%, Figs. 1, 2A, Supplementary Data 1: folder 1), despite having some of the lowest *T*_*b*_ and BMR among mammals^20^. In addition, sloths have low BMR and also face significant body temperature variations^46^. Mammals that undergo large body temperature variations severely decrease their metabolic rates, in relation to torpor are referred to as heterothermic^24,25^. As a result, their turbinals could act to minimize heat/moisture loss. However, our results point out that irrespective of different forms of heterothermy, there is no relation to the Maxillo RSA. Given our results, we may infer that the Maxillo RSA and the maxilloturbinal complexity may be influenced by other factors unrelated to metabolism, body temperature, or heterothermy.

Alternatively, there may be a potential trade-off between maxilloturbinal and nasoturbinal or even with other structures such as the trachea^16^. However, based on the dataset from Martinez et al.^18^ we demonstrated in 132 species that there is no trade-off between the size-corrected surface area of the maxillo- and the nasoturbinal (Fig. Supplementary Fig. 7). In addition, due to the known variation of the nasoturbinal epithelial cover among mammals^33,35,37–39^ and the scarcity of the data on trachea, it will be extremely challenging to undertake at the scale of mammals. Also, there is an important caveat because such work would be based on soft tissues that will not be comparable to extinct species except by ancestral state reconstructions. Other bony structures seem to hold promise, such as the bony labyrinth and the morphofunction of its canals, which has recently been proven to be a precise indicator of endothermy”^9^.

### Could there be a link between environmental conditions and maxilloturbinal surface area?

It is plausible to hypothesize that maxilloturbinal morphology may be related to environmental conditions to which the species is adapted. The maxilloturbinal function could have a more prominent heat/moisture exchange role in species that face harsh environmental conditions, thus helping to limit spurious heat and moisture loss. For example, the species with the second highest predicted Maxillo RSA (463%) is *Rangifer* (Figs. 1, 2A, Supplementary Data 1: folder 1), which is arctic species, known to have efficient heat and moisture conservation capacities^47^. However, in Carnivora, the density of the maxilloturbinal within the nasal chamber does not seem associated with species living in arid or cold habitats^48^. When species are obliged to face harsh thermal or water-stress conditions and do not resort to torpor to avoid them, they may have to rely on a well-developed, proportionally large, complex maxilloturbinal. Following this hypothesis, it has been found that amphibious and aquatic mammals have among the highest values of Maxillo RSA^17,18^. This pattern had been interpreted as an adaptation to restrict heat loss due to the high thermal inertia of water^17,18^. Here, we have shown that this pattern for increasing Maxillo RSA in amphibious and aquatic species is convergent across mammals (Figs. 1, 2A, Supplementary Data 1: folder 1). Moreover, the increase of Maxillo RSA in amphibious and aquatic species is also associated with an increase in turbinal complexity (Fig. 4). For example, in monotremes, the maxilloturbinal complexity between the amphibious *Ornithorhynchus* and the terrestrial *Tachyglossus* strongly differs (Fig. 4). The platypus has a very complex maxilloturbinal with several small lamellae originating from the main three branches, being similar to some aquatic or amphibious species as well as to some Carnivora (Fig. 4, e.g.,^17,18^). In contrast, *Tachyglossus* has no additional lamellae to the main three branches but a thick maxilloturbinal (Fig. 4).

Lastly, some studies suggested or described some potential positive relation between the maxilloturbinal and temperature and/or altitude (ref. ^17,48–50^ and supplementary results in^18^). However, this pattern needs to be properly tested with a specific sampling that captures ecological adaptations and the effects of phylogenetic inertia.

### Water conservation

Another major role of the maxilloturbinal is water conservation that on average allows individuals to conserve two-thirds of the humidity of the exhaled air^1,3,10–15^. Experimental studies have demonstrated the importance of nasal breathing and respiratory turbinals in water conservation. Indeed, mammals with plugged nares that are forced to breathe through the mouth, significantly increase evaporative water loss (EWL^3^). *Heterocephalus glaber* appears to avoid breathing through the mouth when performing energy intensive digging because the lips close behind the digging incisors (named inflexa pellita)^43,51^, and this species has the lowest value of predicted Maxillo RSA (6%, Figs. 1, 2A, Supplementary Data 1: folder 1) of the entire sample. In birds, when respiratory turbinals are lost or reduced, their longer trachea can compensate for heat and moisture conservation^16^. Apparently, this is not the case with *H. glaber* that has the highest EWL recorded in mammals (ref. ^41^ but see also^52^). As an example, in experimental conditions, the naked mole-rat has a water evaporation rate up to 10 times higher than *Gerbillus pusillus*, a terrestrial rodent of comparable body mass that co-occurs aboveground in the same habitat^41,52^. Since *H. glaber* lives underground in relatively humid burrows (31.2% to 92.8%^53^), it may not need to conserve as much water as *G. pusillus* and, therefore, to have large maxilloturbinals. Extensive literature exists about water conservation in species that live in arid regions^12–14,47,54,55^. As an example, the Kangaroo rats (*Dipodomys spectabilis*) that live in hot and desert environments are known to have higher water conservation capacities than other rodents living in temperate habitats^12^. However, these studies generally considered the nasal cavity as a whole.

We showed that marine mammals have developed maxilloturbinals to limit heat loss (see above). As an example, *Mirounga* and *Zalophus* have extremely complex and well developed maxilloturbinals (Figs. 1, 2A, Supplementary Data 1: folder 1 and ref. ^17,56^). This may be also associated with efficient water conservation capacities resulting in an adaptation to a salty environment^57^. However, data on EWL with comparable experimental design are lacking to properly test the relation between maxilloturbinal and EWL at large.

### A multifactorial physiological question

We demonstrated the absence of relation between the Maxillo RSA and some mammalian physiological traits, such as metabolism, body temperature, and heterothermy. As this is the case with olfaction, and therefore, olfactory turbinals^58^, the relation between thermal biology and maxilloturbinal is driven by multifactorial processes. For example, other factors may influence the absence of relation such as the body surface evaporation^59^, the efficiency of oxygen extraction^54^, the efficiency of renal mechanism for water conservation^60^, as well as the lung structure^57^.

Further studies may address the role of the maxilloturbinal in the light of: (1) their relation with the nasoturbinal as well as with the overall nasal cavity (e.g., including unossified structures such as the atrio- and marginoturbinals), as well as with (2) their epithelium and the gap width^14,61,62^.

In addition to their role in heat and moisture conservation, respiratory turbinals play a role in other functions that may also have driven their evolution. For example, respiratory turbinals redirect the inspired airflow to specific areas^63–65^, and have a protective role against toxic and abrasive elements during inspiration^10,66,67^. Finally, the potential role of respiratory turbinals in brain cooling via the carotid rete has also been discussed^16,55,68–70^ but received comparatively little attention over the past few years.

## Methods

### Data acquisition

424 individuals with undamaged maxilloturbinal belonging to 310 mammal species were selected from museums (Supplementary Data 1: folder 1) and scanned using high-resolution X-ray micro-computed tomography (µCT). The use of museum specimens was carried out in accordance with the relevant permissions and ethical approvals of the different museums. Of the 424 individuals, 32 were downloaded from Morphosource^71^ and 6 from DigiMorph (Supplementary Data 1: folder 1). The left maxilloturbinal was segmented following Martinez et al.^18,58,72^ (Supplementary Fig. 8) with AvizoLite 2020.1 (VSG Inc.). When the left maxilloturbinal was damaged we used the right maxilloturbinal. Using the brush tool, the bony part of the maxilloturbinals were manually segmented (Supplementary Figs. 8, 9, 10) in approximately one in five images. The segmentation was then interpolated with Avizo then, all images were checked and the errors were manually corrected. For the interpolation, the number of unsegmented images between the segmented images varies according to the complexity of the maxilloturbinal as well as the quality of the image that is not exclusively associated with the resolution (e.g., noise, sharpness and contrasts). In our segmentation we only considered the maxilloturbinal and did not segment other turbinals that may be located in the same area (e.g., ethmoturbinal I, nasoturbinal, semicircular lamina; Supplementary Fig. 9). In addition to mammalian skulls, some unstained ethanol-preserved heads were CT-scanned. With high quality CT data, there is no difficulty to only segment the bony part of the maxilloturbinal from these ethanol-preserved heads. However, in the case of old specimens (e.g., when the epithelium dried) or with CT data of low quality, the delimitation between the epithelium and the bony structure may be difficult and generally result in overestimation (e.g., in the case of CT data of low quality). For these reasons we generally excluded CT data of low quality. In the very few cases where we were obligated to use such data (e.g., rare species where very few data are available) particular attention has been paid to only select the bony part (e.g., in *Ornithorhynchus*; Supplementary Fig. 10). In order to follow a highly consistent segmentation and extract accurate quantitative data, all individuals were similarly segmented. In some species the maxilloturbinal is attached to the nasolacrimal canal (and therefore to the lamina infraconchalis) and then posteriorly split off from it (Supplementary Fig. 8). In this case and during the split off, we only segmented the maxilloturbinal (Supplementary Fig. 8). The posterior end of the maxilloturbinal may also be tricky to delimit in some species since it is connected to an additional ridge that posteriorly forms the lateral wall of the nasopharyngeal duct. We carefully excluded this additional ridge from our segmentation (Supplementary Fig. 8). For CT data based on mammalian skulls, it may also be difficult to check with confidence that the most anterior part of the maxilloturbinal is not broken. For this, the skulls were carefully selected before being CT-scanned as well as later in Avizo. In addition, all the segmentations were performed by the same operator (Q.M.) who has experience with turbinal bones. In some cases, if there is a doubt, the status of the maxilloturbinal was checked with CT data based on ethanol-preserved head. Finally, in some species we segmented several individuals (424 individuals from 310 species) to limit potential artifacts.

### Metabolic rate, body temperature and ventilation rate

Maxilloturbinal surface areas were standardized by skull length. The named “*relative surface area of maxilloturbinal*” (Maxillo RSA) is based on the log-log residuals of the phylogenetic generalized least squares (PGLS) regression between maxilloturbinal surface area and skull length (Figs. 1, 2A, Supplementary Data 1: folders 1, 2, 3). This was performed with the *gls* function from the R package nlme^73^ in R^74^. To avoid negative values, we added the lowest residual value (2.77, Supplementary Data 1: folders 1, 2, 3) to all residuals. We used the following equation to estimate how Maxillo RSA deviates from the prediction (see also Supplementary Data 1: folders 1, 2, 3): (e^[(residuals of the model)+(prediction of the model)]*100)/e^(prediction of the Model). The model is the PGLS regression between maxilloturbinal surface area and skull length. The predicted values were obtained with the *Predict* function from the R package car^75^ and were named “*Predicted Maxillo RSA*”. Mammalian basal metabolic rates (BMR, in watt), body temperatures (*T*_*b*_), and body mass (bm) were extracted from Clarke et al. ^76^. To limit the effect of size, we used the corrected basal metabolic rate (cBMR), which corresponds to the residuals of the log-log PGLS between BMR and bm. We performed PGLS between cBMR and Maxillo RSA (Fig. 2B) and between *T*_*b*_ and Maxillo RSA (Fig. 2C). Following Araújo et al.^9^ we defined an endotherm as an animal producing heat throughout its entire body via metabolism (not shivering and/or muscular thermogenesis), maintaining nearly constant body temperature largely independently from external conditions, and excluding phases of short-term torpor, aestivation, and hibernation. Following this definition, it was demonstrated that *T*_*b*_ is a valid proxy for endothermy^9^.

An alternative measure to heat and water loss from the respiratory tract may be the ventilation rate (ml.min-1) that corresponds to: tidal volume (ml) x breathing rate (min-1). We performed linear regression between the ventilation rate and Maxillo RSA (Supplementary Data 1: folders 1, 8). Tidal volume and breathing rate were extracted from Stahl^77^ and Frappell et al.^78^ and transformed to obtain ventilation rate in ml.min-1. Because ventilation rate data greatly differ between studies, we performed three different linear regressions: (1) with all the data merged between Stahl^77^ and Frappell et al.^78^, (2) only with the data from Stahl^77^, (3) only with the data from Frappell et al.^78^. Mean was performed when a species was present in the two studies. The normality of the data was tested on the residuals of the linear model with the Shapiro-Wilk’s test and the function *shapiro.test* from the *stats* r package^74^. The heteroscedasticity of the linear model was tested with the Breusch-Pagan test and the function *bptest* from the *lmtest* r package^79^. When we could assume the normality and the homogeneity of the data, the potential correlation was tested with the function *lm* and *summary* from the *stats* r package^74^. When the normality and/or the homogeneity was rejected, the potential correlation was tested with the nonparametric Spearman’s rank correlation using the function *cor.test* from the *stats* r package^74^ (Supplementary Fig. 5).

For Maxillo RSA, to avoid negative values, we added the lowest residual value, 2.64 and 2.67 respectively (Supplementary Data 1: folders 1, 2, 3). These two PGLS comprised 99 and 89 species respectively. We also performed PGLS and linear regressions between cBMR and the relative surface area of the maxilloturbinal based on body mass (Supplementary Fig. 3A) as well as between *T*_*b*_ and the relative surface area of the maxilloturbinal based on body mass (Supplementary Fig. 3 B). The phylogenetic signal of the Maxillo RSA was calculated with Blomberg’s K^80^ and Pagel’s lambda^81^ with the *phylosig* function from the R package phytools^82^ (Supplementary Data 1: folder 4). The phylogenetic figures were generated by the R package phytools^82^. We used a maximum clade credibility (MCC) phylogeny obtained from 10,000 trees sampled in the posterior distribution of^83^ and pruned to match the species in our dataset. The MCC consensus tree was inferred using TreeAnnotator v.1.8.2^84^ with a 25% burn-in. To limit the number of ecological variables, amphibious and aquatic species were labeled “amphibious” (Figs. 1, 2).

### Ecology

Although our dataset was not designed to test ecological lifestyle (e.g., we did not sample all the independent lineages for a given ecology), we tested it to have a general idea on the potential relation between the ecology and the Maxillo RSA. Five ecological categories were defined as follows: terrestrial, arboreal, amphibious, subterranean and flying (Fig. 1, Supplementary Data 1: folders 1, 2, 3, 5, 6, 7). The normality and the homogeneity of the data were tested as described above. When we could assume the normality and the homogeneity of the data, we performed an ANOVA on the PGLS model including the tested variables, as well as the ecology and their potential interactions. We also performed ANOVA between a single variable and the ecological data. This was performed with the function *anova* from the *stats* r package^74^. When we could not assume the normality and/or the homogeneity of the data, we performed the non-parametric Kruskal-Wallis test to test the variables as well as the ecology and their potential interactions (Supplementary Table 1). This was performed with the function *kruskal.test* from the *stats* r package^74^. We similarly tested the impact of heterothermy on the Maxillo RSA with a first test including two categories (no heterothermy, heterothermy) and a test including four categories (no heterothermy, hibernation, aestivation, other; Supplementary Data 1: folders 1, 3). In order to test the significance of only using the maxilloturbinal surface area, we performed PGLS and linear regressions between maxilloturbinal and nasoturbinal surface area with data extracted from Martinez et al.^18^ and based on 132 species (Supplementary Fig. 7).

### Maxilloturbinal complexity

We described the different patterns of turbinal complexity. To date, the increasing turbinal complexity is described as the development of infolding and small lamellae called epiturbinals and resulting from repetitive appositional bone growth^33,85^. From a statistical perspective, turbinal complexity is often described as the degree of details in a predefined area^72,86–88^. Several studies based on fluid dynamic principles have improved our understanding of the functional role of turbinals demonstrating for example that the increase in turbinal complexity increases the proportion of air in contact with mucus gland and epithelium^63,64,86,89^. Therefore, the increase in turbinal complexity may facilitate heat and moisture conservation performances. In rodents, it has been demonstrated that there is a significant correlation between respiratory turbinal complexity and surface area^72^. These results support the functional significance of most turbinal studies that only used the surface area proxy.

## Supplementary information

Supplementary Information

Description of Additional Supplementary Files

Supplementary Data 1

## Data Availability

All the raw data used to perform the analyses are available in the Supplementary Data 1: folder 1. The list of the µCT data is available in the Supplementary Data 1: folder 1. Data owned by the co-authors are available on MorphoSource (https://www.morphosource.org/catalog/media?locale=en&q=quentin+martinez&search_field=all_fields&sort=system_create_dtsi+desc) and on request from the relevant holding institution (see details in the Supplementary Data 1: folder 1: All_Files.xlsx).

## Code availability

All the codes, R script, CSV files and nexus phylogeny needed to perform analyses and figures presented in this study are available in the Supplementary Data 1: folders 1 to 8.
