# Supplementary material for: Mammalian maxilloturbinal evolution does not reflect thermal biology
Source: Nat Commun. 2023 Jul 21;14:4425. doi: 10.1038/s41467-023-39994-1 (PMC10361988; doi:10.1038/s41467-023-39994-1)
Supplement: Supplementary file 1 — Supplementary Information [file 41467_2023_39994_MOESM1_ESM.pdf]

Supplementary Materials for:

Mammalian maxilloturbinal evolution does not reflect thermal biology

Quentin Martinez*, Jan Okrouhlík, Radim Šumbera 3, Mark Wright, Ricardo Araújo, Stan Braude, Thomas B. Hildebrandt, Susanne Holtze, Irina Ruf, and Pierre-Henri Fabre

*Corresponding author.
Email: quentinmartinezphoto@gmail.com

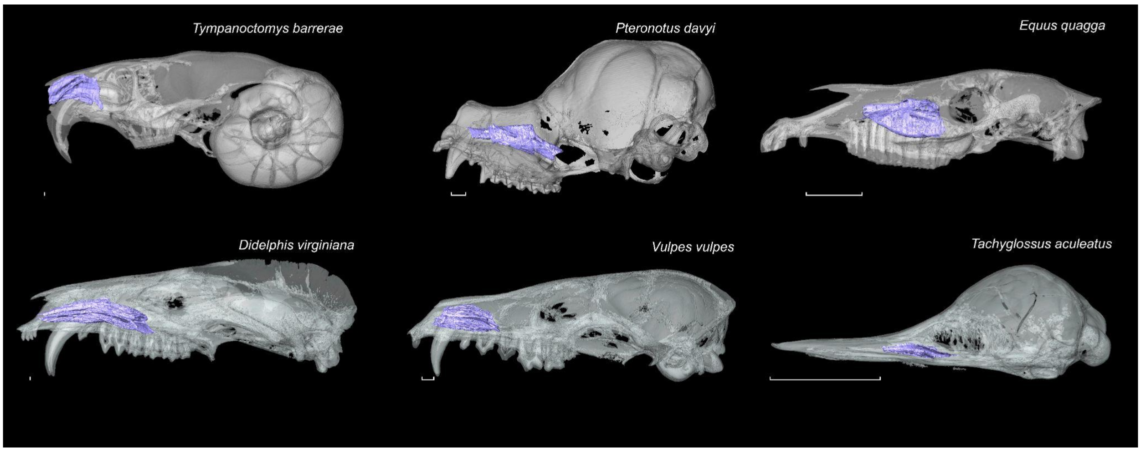

Supplementary Figure 1.
The gap between the anterior part of the maxilloturbinal and the nasal aperture varies considerably among mammals. Not to scale.

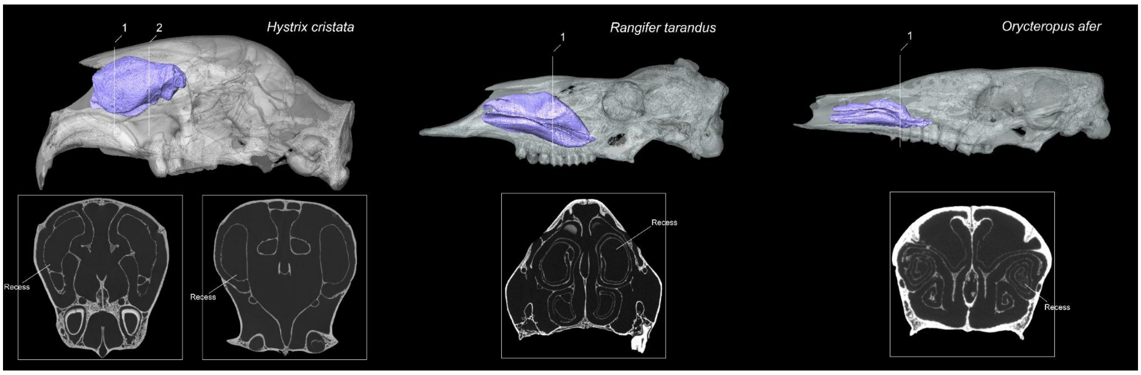

Supplementary Figure 2.
The maxilloturbinal of selected mammalian species forms a recess. The recess was not segmented/considered in the quantitative analysis, only the bony part. Not to scale.

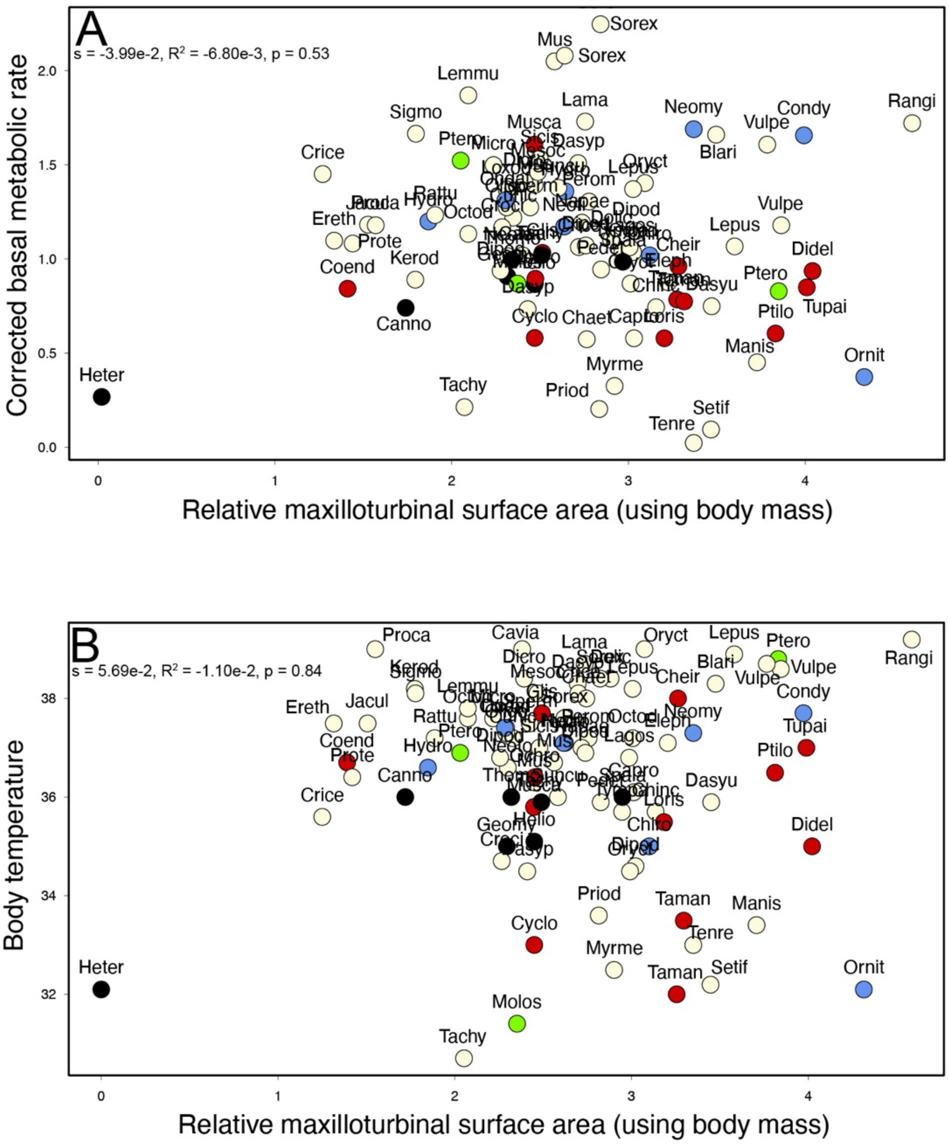

Supplementary Figure 3.

(A) Linear regression between corrected basal metabolic rates (cBMR) and the relative surface area of the maxilloturbinal based on body mass. (B) Linear regression between body temperatures (Tb) and the relative surface area of the maxilloturbinal based on body mass. Barplots: beige = terrestrial, red = arboreal, blue = amphibious, black = subterranean, and yellow = flying species.

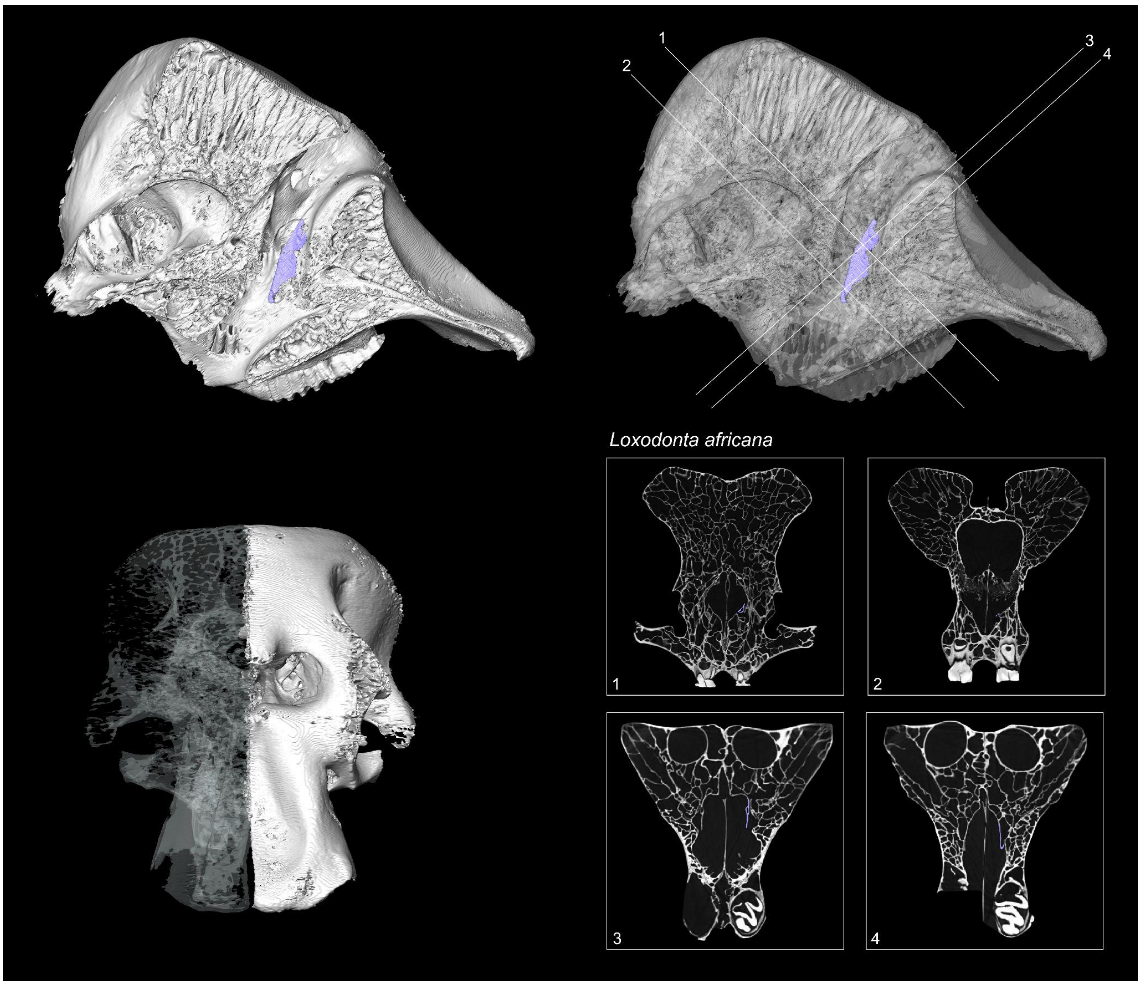

Supplementary Figure 4.
Detailed view of the maxilloturbinal of the African bush elephant (*Loxodonta africana*). Not to scale.

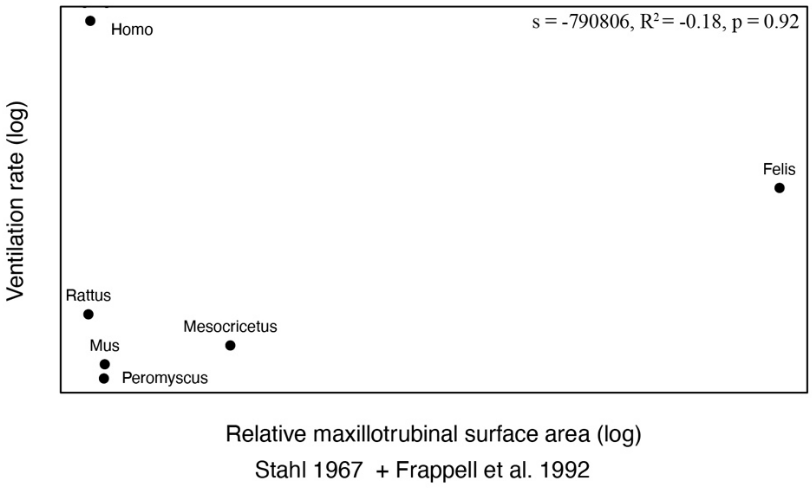

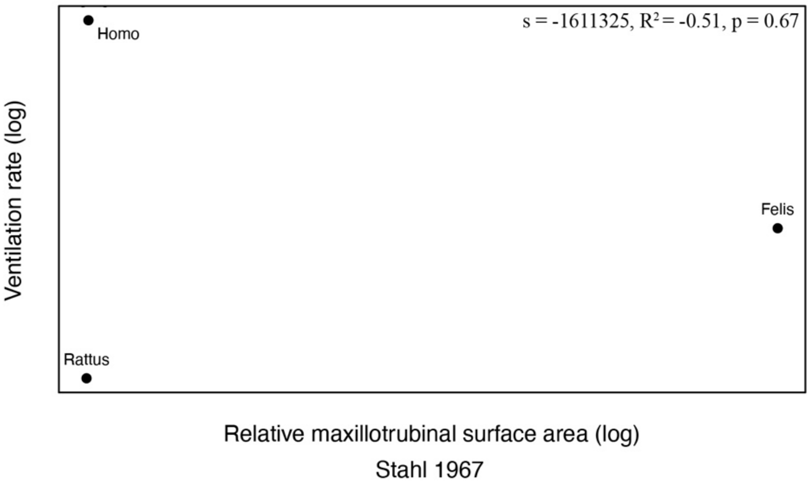

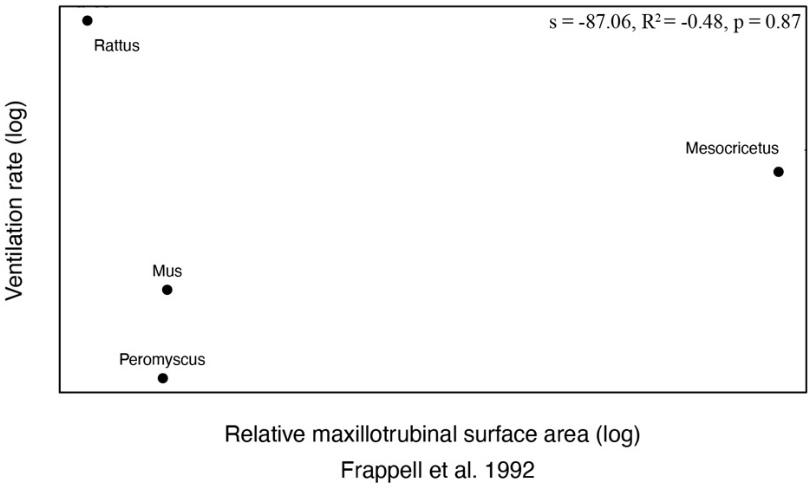

Supplementary Figure 5.
Linear regression between the ventilation rate (log) and Maxillo RSA (log), with: (1) all the data merged between (Stahl 1967) and (Frappell et al. 1992), (2) only the data from (Stahl 1967), (3) only the data from (Frappell et al. 1992).

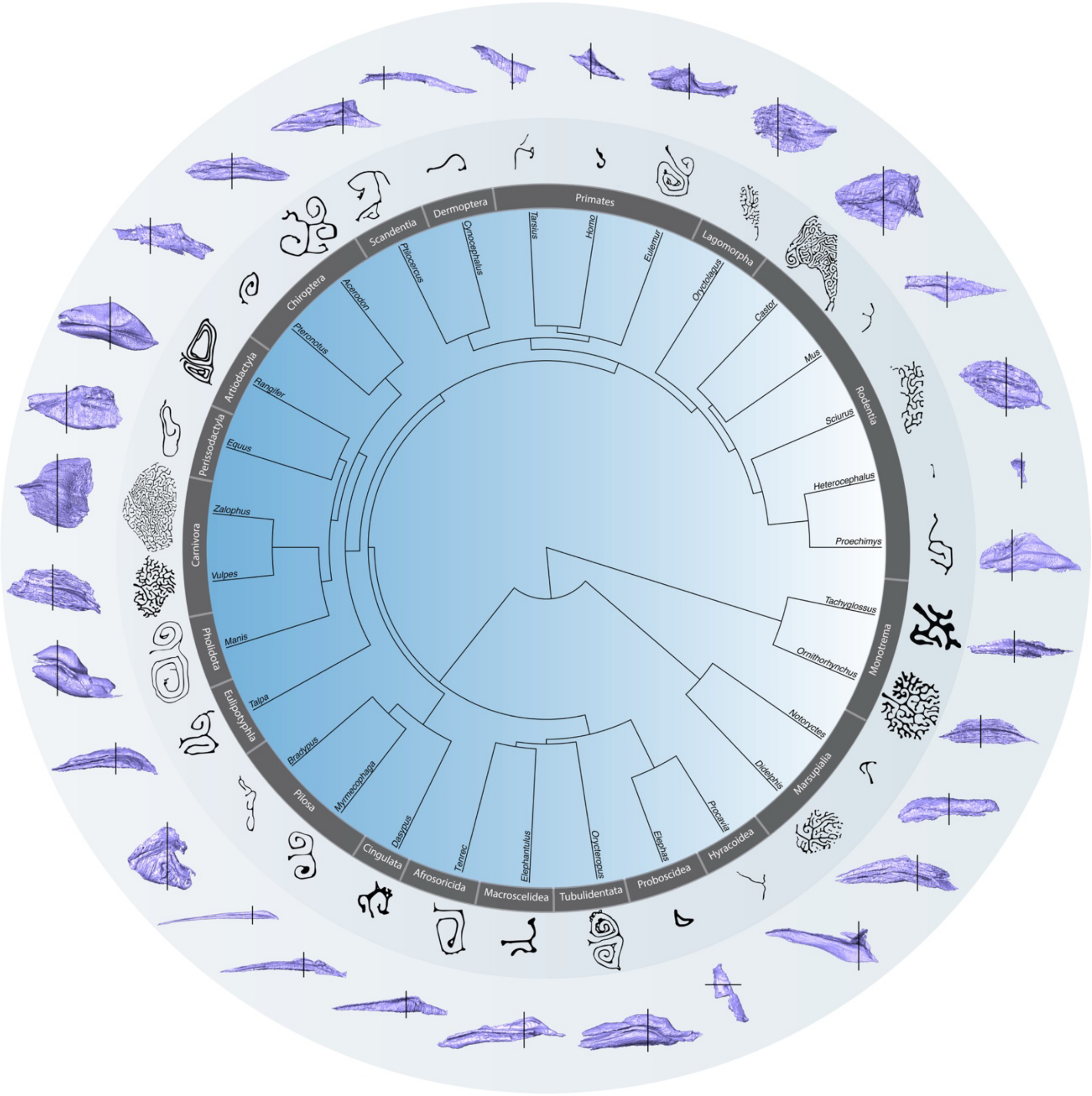

Supplementary Figure 6.

Silhouettes of the most complex area of the maxilloturbinal coronal cross section across mammalian major clades and 3D representations of the maxilloturbinal. The black vertical bar represents the most complex area used to illustrate the turbinal complexity.

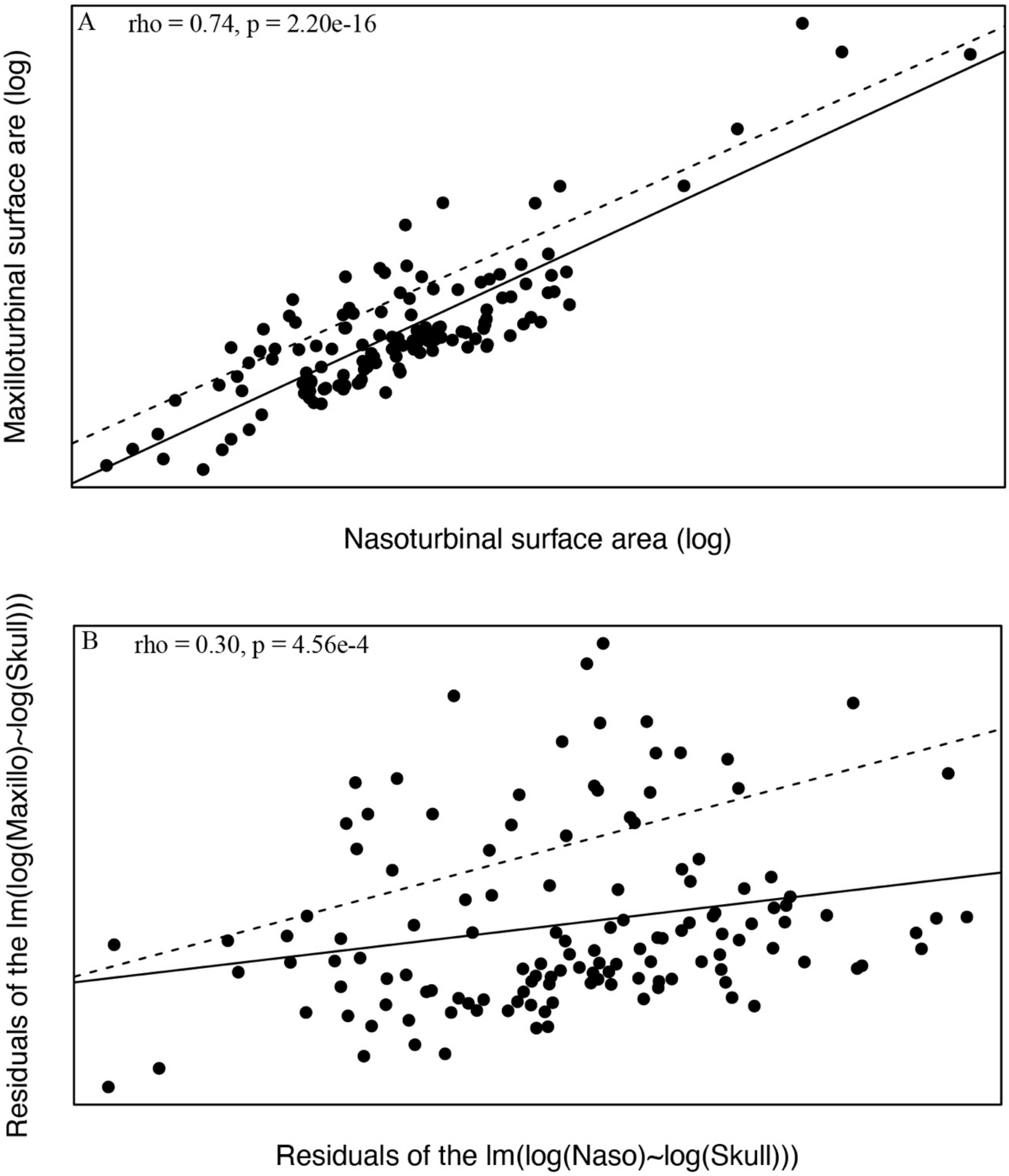

Supplementary Figure 7. Absence of trade-off between the size-corrected maxilloturbinal and the nasoturbinal.

A. Log–log regression (continuous line) and PGLS (dashed line) of maxilloturbinal surface area on nasoturbinal surface area (left). **B**. Regression (continuous line) and PGLS (dashed line) of the residuals of the linear model between maxilloturbinal surface area and skull length as well as between nasoturbinal surface area and skull length (right). Plot B indicates that the variation observed in the corrected maxilloturbinal surface area significantly correlates with the variation observed in the corrected nasoturbinal, suggesting an absence of a trade-off. The data were extracted from Martinez et al. (2020) and tested with the Spearman's rank-correlation test.

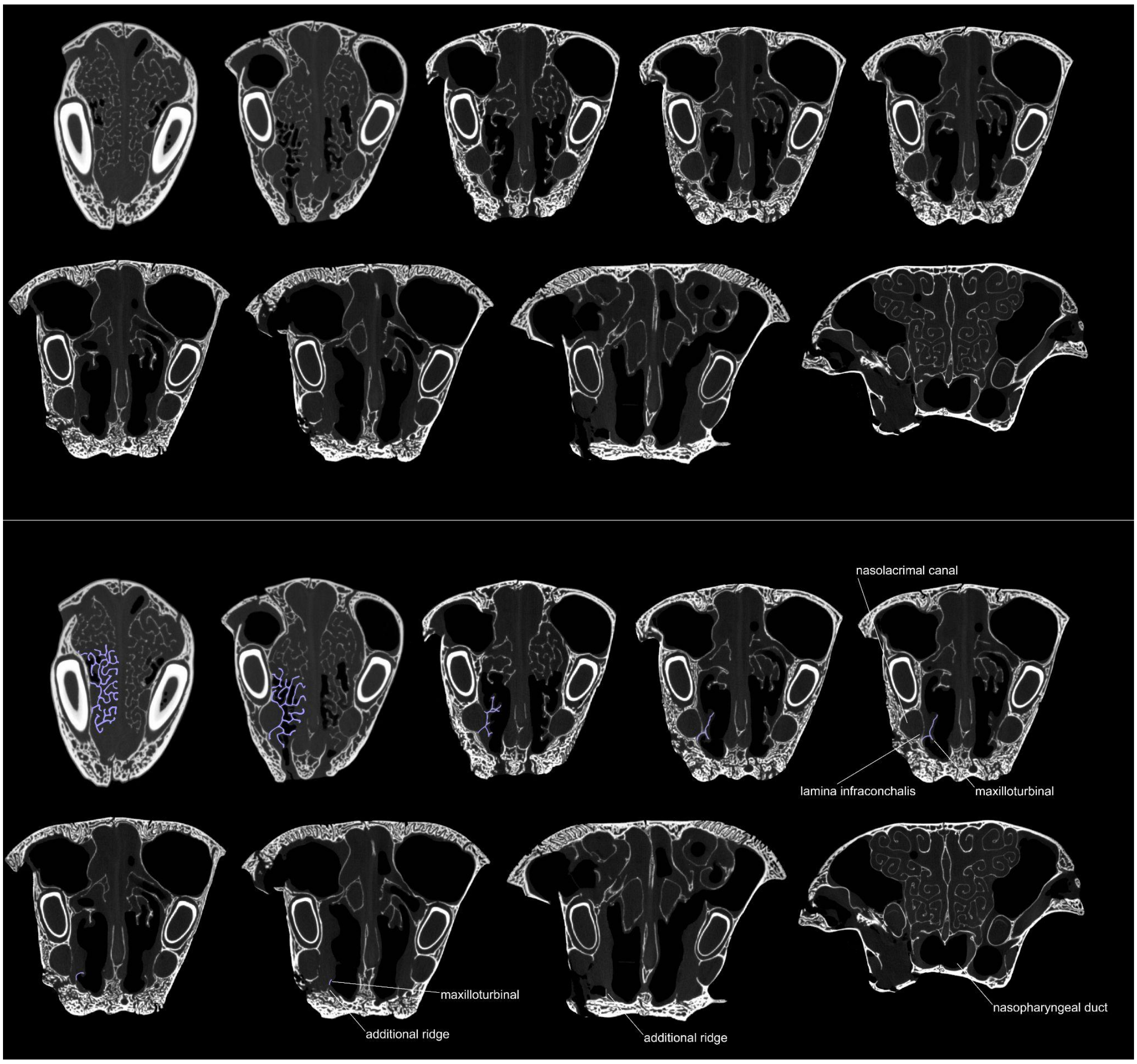

Supplementary Figure 8. Example of maxilloturbinal segmentation.

Detailed view of the maxilloturbinal in *Sciurus vulgaris* showing how we consistently segmented it (see explanations in the Materials and Methods section). The maxilloturbinal drawing in the coronal views does not represent the actual segmentation thickness and only illustrates the selected area of the maxilloturbinal.

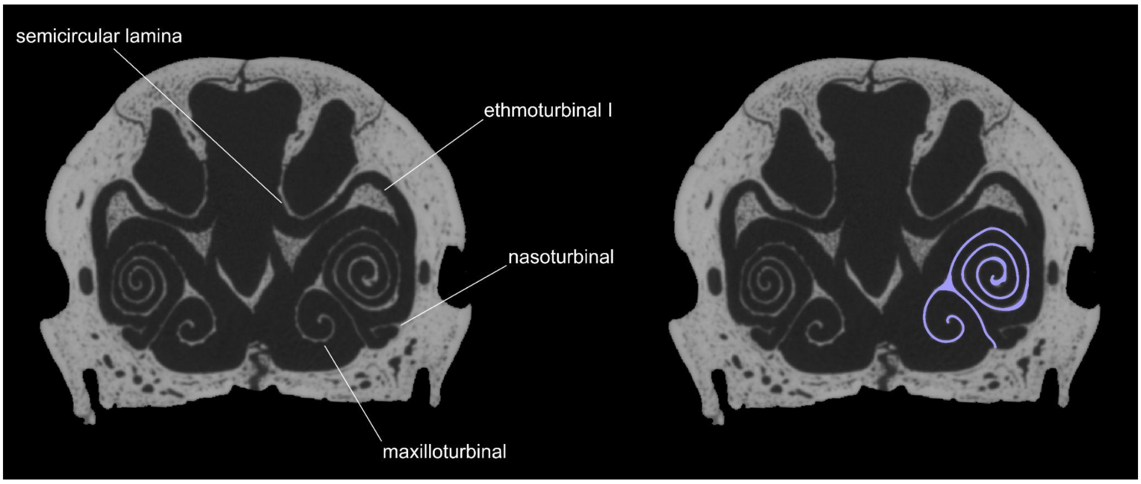

Supplementary Figure 9. Example of maxilloturbinal segmentation.
Detailed view of the maxilloturbinal in *Priodontes maximus* showing how we only segmented it (see explanations in the Materials and Methods section). The maxilloturbinal drawing in the coronal views does not represent the actual segmentation thickness and only illustrates the selected area of the maxilloturbinal.

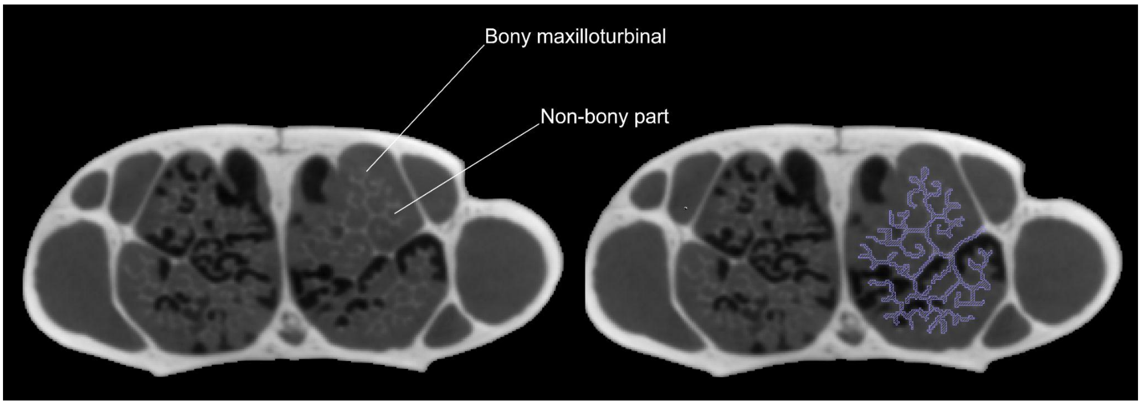

Supplementary Figure 10. Example of maxilloturbinal segmentation.
Screenshot of the Avizo segmentation in *Ornithorhynchus anatinus* showing that a particular attention has been paid to only select the bony maxilloturbinal when we were obligated to use CT data of low quality (see explanations in the Materials and Methods section).

Supplementary Table 1.

Testing the impact of the ecology and the interactions

Variable	Testing	p-value	test
Maxilloturbinal surface area	Ecology	**0.05**	Kruskal
Maxillo RSA	Ecology	**3.40e 10-4**	one-sided Anova
Maxillo RSA based on body mass	Ecology	**0.05**	one-sided Anova
Skull length	Ecology	**0.02**	Kruskal
Tb	Ecology	**0.03**	Kruskal
cBMR	Ecology	**0.01**	Kruskal
Interaction Maxilloturbinal surface area and Ecology	to Skull length	0.49	Kruskal
Interaction Skull length and Ecology	to Maxilloturbinal surface area	0.47	Kruskal
PGLS (cBMR ~ Maxillo RSA)	Ecology	0.65	two-sided anova (PGLS)
Interaction with Ecology	Maxillo RSA and cBMR	0.33	two-sided anova (PGLS)
PGLS (cBMR ~ Maxillo RSA based on body mass)	Ecology	0.62	two-sided anova (PGLS)
Interaction with Ecology	Maxillo RSA and cBMR	0.13	two-sided anova (PGLS)
Interaction Teb and Ecology	to Maxillo RSA	0.32	Kruskal
Interaction Maxillo RSA and Ecology	to Tb	0.48	Kruskal
Interaction maxillo RSA based on body mass and Ecology	to Tb	0.48	Kruskal
Interaction Tb and Ecology	to maxillo RSA based on body mass	0.25	Kruskal
Interaction cBMR and Ecology	to Maxillo RSA	0.48	Kruskal
Interaction Maxillo RSA and Ecology	to cBMR	0.48	Kruskal
Interaction Maxillo RSA based on body mass and Ecology	to cBMR	0.48	Kruskal
Interaction cBMR and Ecology	to Maxillo RSA based on body mass	0.48	Kruskal
