# Supplementary material for: Mammalian maxilloturbinal evolution does not reflect thermal biology
Source: Nat Commun. 2023 Jul 21;14:4425. doi: 10.1038/s41467-023-39994-1 (PMC10361988; doi:10.1038/s41467-023-39994-1)
Supplement: Supplementary file 2 — Description of Additional Supplementary Files [file 41467_2023_39994_MOESM2_ESM.pdf]

Description of Additional Supplementary Files

File Name: Supplementary Data 1
Description: R script, CSV files and nexus phylogeny needed to perform analyses and figures presented in this study.

Inventory of the Supplementary Data 1:

Mammalian maxilloturbinal evolution does not reflect thermal biology

Quentin Martinez*, Jan Okrouhlík, Radim Šumbera, Mark Wright, Ricardo Araújo, Stan Braude, Thomas B. Hildebrandt, Susanne Holtze, Irina Ruf, and Pierre-Henri Fabre

*Corresponding author.
Email: quentinmartinezphoto@gmail.com

Folder_1_CSV_and_XLSX_files
All CSV and XLSX files including all the raw data needed to perform analyses and figures.

Folder_2_Figures_and_SI:
R scripts used to perform the figures and the supplementary figures.

Folder_3_Heterothermy
R scripts, CSV file and nexus phylogeny to test the heterothermy.

Folder_4_Pagel_lambda
R script to test the phylogenetic inertia.

Folder_5_Phylogenetic_tree
Nexus phylogeny of the sampled species.

Folder_6_Stats_interaction
R scripts, CSV files and nexus phylogeny to test the ecology and their interactions.

Folder_7_Tradeoff_MaxilloNaso
R script, CSV file and nexus phylogeny to plot and test the relation between maxillo and nasoturbinals as well as the potential trade-off.

Folder_8_Ventilation_rate
R scripts and CSV files to test the relation between maxilloturbinal and the ventilation rate.
